# Use of the Capture-Recapture Method to Estimate the Frequency of Community- and Hospital-Acquired Drug-Induced Acute Kidney Injuries in French Databases

**DOI:** 10.3389/fphar.2022.899164

**Published:** 2022-07-05

**Authors:** Amayelle Rey, Valérie Gras, Julien Moragny, Gabriel Choukroun, Kamel Masmoudi, Sophie Liabeuf

**Affiliations:** ^1^ Division of Clinical Pharmacology, Pharmacoepidemiology Department, Amiens University Hospital, Amiens, France; ^2^ MP3CV Laboratory, EA7517, Jules Verne University of Picardie, Amiens, France; ^3^ Division of Nephrology, Amiens University Hospital, Amiens, France

**Keywords:** acute kidney injury (AKI), drugs, frequency, capture—recapture, database

## Abstract

Drug-induced acute kidney injury (AKI) can occur both in primary care (i.e., community-acquired AKI (CA-AKI)) and in hospital settings (i.e., hospital-acquired AKI (HA-AKI)). The reported prevalence of these events varies markedly from one study to another, mainly due to differences in the study design. To estimate the frequency of drug-induced AKIs (both CA-AKIs and HA-AKIs) observed in a French university hospital, we applied the capture-recapture method to 1) the French national pharmacovigilance database (FPVD) and 2) a cohort of hospitalized patients with drug-induced AKIs (documented by analyzing the French national hospital discharge database and the patients’ electronic medical records). Drug-induced AKIs were determined according to the Naranjo algorithm and then categorized as CA-AKIs or HA-AKIs. A total number of 1,557 episodes of AKI were record during the study period, of them, the estimated total number of drug-induced AKIs was 593 [95% confidence interval (CI): 485–702], and the estimated prevalence was 38.1% [95%CI: 35.67–40.50]. The prevalences of HA-AKIs and CA-AKIs were similar (39.4% [36.24–42.54] and 37.4% [33.67–41.21], respectively). Only 6.1% of the drug-induced AKIs were recorded in the FPVD, and the proportions of recorded HA-AKIs and CA-AKI differed markedly (3.0% vs. 10.5%, respectively). One of the most frequently involved drug classes were antibiotics in the HA-AKI subgroup (13.0%) and antineoplastics in the CA-AKI subgroup (8.3%). Application of the capture-recapture method to two incomplete data sources can improve the ability to identify and quantify adverse drug reactions like AKIs. The frequency of drug-induced AKI is relatively high and is probably underestimated. The clinical management of an AKI might depend on where it originated.

## 1 Introduction

Acute kidney injury (AKI) is a common complication in hospitalized patients. The aetiology of AKI is very often multifactorial, with several precipitating factors (Robert et al., 2019). Drugs constitute one of the leading causes of AKI (Pierson-Marchandise et al., 2017), with a prevalence ranges from 8% to 60%, depending on the study (Khajavi Rad et al., 2017). Drug-induced AKIs can occur both in primary care (i.e., community-acquired AKI (CA-AKI)) or in hospital settings (i.e., hospital-acquired AKI (HA-AKI)) (Robert et al., 2019). The prevalence of both types of drug-induced AKI varies greatly from one study to another—mainly due to differences in study designs and event definitions, leading to potential bias. Drug-induced AKI constituted the third to fifth leading cause of HA-AKI (Kane-Gill and Goldstein, 2015) and accounted for 37.5% of all HA-AKIs in a study in China (Liu et al., 2020), whereas Robert et al.’s study in France found that drug-induced AKI accounted for 58.8% of all CA-AKIs (Robert et al., 2019). Other researchers have reported prevalence between 59.9% and 72% (Hsu et al., 2007; Wang et al., 2017). To the best of our knowledge, very few studies have estimated and compared the prevalence of drug-induced CA-AKI and HA-AKI in the same cohort.

Although adverse drug reactions (ADRs, including drug-induced AKIs) represent a major source of morbidity among inpatients, their extent remains difficult to study. Indeed, drug safety monitoring is mainly based on spontaneous ADR reporting. This process is limited, however, by under-reporting: it is thought that only 5%–10% of ADRs are notified to the authorities, which makes it difficult to interpret incidences or accurately evaluate the ADRs’ impact (Bégaud et al., 2002). Chart review can identify a high proportion of the ADRs occurring in hospital but is time-consuming and difficult to perform on a routine basis. Hospital administrative databases like the French national hospital discharge database (*Programme de Médicalisation des Systèmes d'Information* [PMSI]) constitute a major source of population-based information and are very useful for conducting safety studies and detecting ADRs (Chun et al., 2019; Osmont et al., 2021). However, the use of these databases alone underestimates the frequency of specific types of ADR, such as drug-induced AKIs. Lastly, none of the various sources of data on ADRs (spontaneous reporting or hospital/administrative databases) is exhaustive.

The capture-recapture method allows estimation of the total number of events or the total size of a population by comparing several information sources (Lugardon et al., 2006). The method was developed by ecologists to evaluate the number of animals in an ecosystem: the animals were captured, marked, released and (for a proportion) recaptured.

The objective of the present study was to estimate the frequency of drug-induced AKIs (i.e., CA-AKI and HA-AKI) in a French university hospital by applying the capture-recapture method to two data sources: 1) the French national pharmacovigilance database (FPVD) and 2) a cohort of hospitalized patients with drug-induced AKIs, constituted by the extraction of data from the PMSI and chart review.

## 2 Materials and Methods

The study was conducted at Amiens Picardie University Hospital (Amiens, France). In 2019, the hospital had 1,664 beds, including 1,238 in medical, surgical and obstetrics wards. A total of 60,134 patients were admitted during the course of 2019, with 289 admitted to the Nephrology and Transplantation intensive care unit (including 82 for kidney transplantation), 2,566 to the cardiothoracic surgery unit, 787 to the intensive care units, and 1,285 to the Nephrology Department.

### 2.1 Data Sources

#### 2.1.1 A Cohort of Hospitalized Patients With Drug-Induced Acute Kidney Injuries

The IRA-PMSI retrospective, single-centre study was performed from 1 January 2019, to 30 June 2019. The goal was to use data extracted from the PMSI and electronic medical records (EMRs) to build a cohort of hospitalized patients having experienced an AKI. The IRA-PMSI study’s objectives and procedures were approved by an independent ethics committee (CPP Nord Ouest II, Amiens, France; reference: RCB 2020-A00556-33), and the study was registered at ClinicalTrials.gov (NCT04923750). The patient selection procedure has been described elsewhere and is summarized in the Supplementary Methods (Rey et al., 2021). The distinction between CA-AKI and HA-AKI was determined after manually screening of each eligible patient’s electronic medical records and laboratory data.

Each eligible patient’s EMRs was manually screened for drug-induced AKIs by an expert pharmacologist, using the Naranjo algorithm (Naranjo et al., 1981). On the basis of 10 questions concerning chronological, semiological and bibliographic criteria, the ADR (i.e., AKI) was assigned to a probability category according to the total Naranjo score, as follows: definite: ≥9; probable: 5 to 8; possible: 1 to 4; doubtful: ≤0. Drugs were eligible if Naranjo’s score was superior to 0. A second expert pharmacologist rated the Naranjo score for 100 randomly selected drug prescriptions; the correlation between the experts’ respective scores was good. Each drug-induced AKI was then categorized as a CA-AKI or an HA-AKI (Supplementary Methods and Supplementary Figure S1) (Rey et al., 2021).

#### 2.1.2 The French National Pharmacovigilance Database

In a second step, we identified cases of drug-induced AKIs having occurred at Amiens Picardie University Hospital during the same period (1 January 2019, to 30 June 2019) and having been spontaneously reported to the regional pharmacovigilance centre (Amiens, France). To do so, we screened the FPVD, which was established in 1985. In France, notification of ADRs to the French pharmacovigilance system is mandatory for healthcare professionals. For each spontaneous notification, were collected data on the patient, the drug exposure, and the event. To identify cases, we used the broad standardized Medical Dictionary for Regulatory Activities (MedDRA) query (SMQ) for AKIs (Supplementary Table S1). This broad SMQ is known to identify cases of AKI correctly (Pierson-Marchandise et al., 2017). Although the reports recorded in the FPVD are anonymized, we had access to the original EMRs archived at the regional pharmacovigilance centre; this enabled us to accurately identify common cases in the two data sources (i.e. AKIs in the hospital cohort that were also recorded in the FPVD).

For each selected record of an AKI, we checked that the case met the Kidney Disease: Improving Global Outcomes (KDIGO) definition (based on creatinine only) and we applied the Naranjo’s scale to each drug recorded in eligible notification.

### 2.2 Statistical Analysis

#### 2.2.1 Descriptive Statistics

Descriptive statistics were used to characterize the study population. Each drug-induced AKI was classified as a CA-AKI or an HA-AKI. The cohort’s patients were divided according to whether or not their AKI was reported to the regional pharmacovigilance centre and so was included in the FPVD. Depending on the data type and distribution, groups were compared using Student’s t test, Welch’s two-sample *t*-test, a chi-squared test, or Fisher’s test.

Drug classes were defined according to the Anatomical Therapeutic Chemical (ATC) classification. For all drug-induced AKIs and in both subgroups (i.e. CA- and HA-AKIs), we considered each ATC level 2 class involved in more than 5% of the AKIs and then listed the three most frequently involved active substances for each of the two data sources. The proportion of AKIs recorded in the FPVD was calculated for each ATC level 2 class and for each drug involved in more than 10 AKIs.

#### 2.2.2 The Capture-recapture Method

**FIGURE 1:**
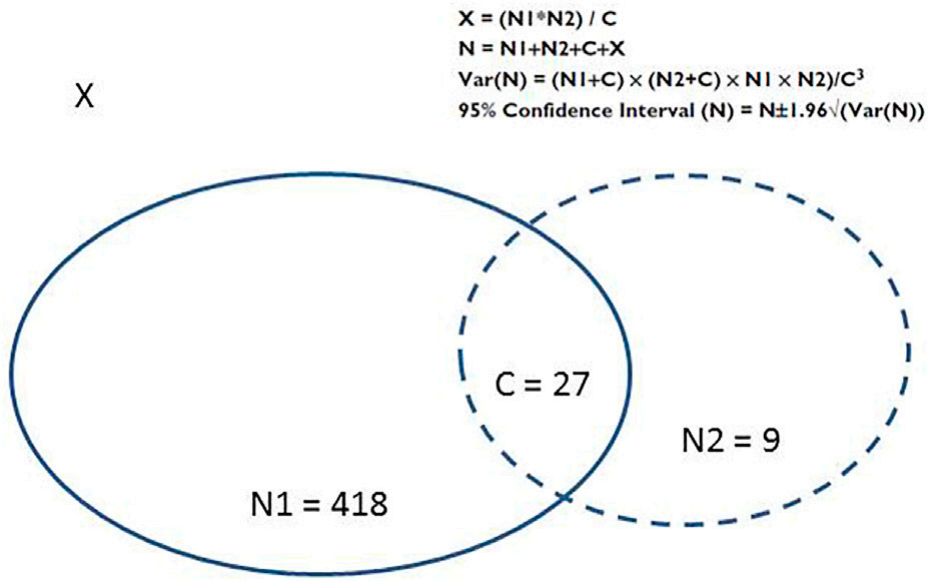
Distribution of cases in the two data sources, and the statistics of the capture-recapture method (Lugardon et al., 2006). C: the number of cases shared by the two sources; N, the total number of cases; N1+ C, the number of AKIs reported by the first information source (i.e., the “capture”); N2 + C: the number of AKIs reported by the second information source (i.e., “recapture”); Source 1: capture (the cohort of patients with drug-induced AKI); Source 2, recapture (the FPVD); Var(N), variance of N; X, the number of unidentified cases.

We used the capture–recapture method to estimate the frequency of drug-induced AKIs overall and the frequencies of drug-induced CA-AKIs and HA-AKIs. By combining the two data sources covering the same patient population, this method estimated the exhaustiveness of monitoring systems and provided the total number of drug-induced AKIs in the population, the variance, the 95% confidence interval (CI), and the number of cases recorded in both data sources (Lugardon et al., 2006). The capture–recapture method’s statistics are summarized in Figure 1.

In the present study, the two incomplete data sources used were the IRA-PMSI cohort (built by selecting only drug-induced AKIs) and the FPVD. Capture was defined as drug-induced AKI identified in IRA-PMSI cohort, and recapture was defined as drug-induced AKI identified in the FPVD. From the estimate of the total number of all drug-induced AKIs, the number of drug-induced AKIs not identified by either of the two sources was deducted, as was the exhaustiveness of the two sources (defined by dividing the number of detected drug-induced AKIs by the estimated total number of drug-induced AKIs). We then calculated the FPVD notification rates for all drug-induced AKIs and the two subgroups (drug-induced CA-AKIs and drug-induced HA-AKIs), using the number drug-induced AKIs notified in the FPVD and the estimated total number of drug-induced AKIs.

Lastly, we checked the capture-recapture method’s validity conditions: 1) all cases of drug-induced AKIs are real, and the case definition is the same for each source; 2) capture in each sources homogeneous; 3) the study population is a closed population; 4) the period and the geographical area are the same for the two data sources; 5) all common cases are identified and are true duplicates; and 6) the data sources are independent.

The threshold for statistical significance was set to *p* < 0.05. All analyses were performed using R software (version 3.7.2, R Foundation for Statistical Computing, Vienna, Austria) (R Core Team (2020), s. d.).

## 3 Results

### 3.1 Case Selection

#### 3.1.1 “IRA-PMSI” Cohort

**FIGURE 2:**
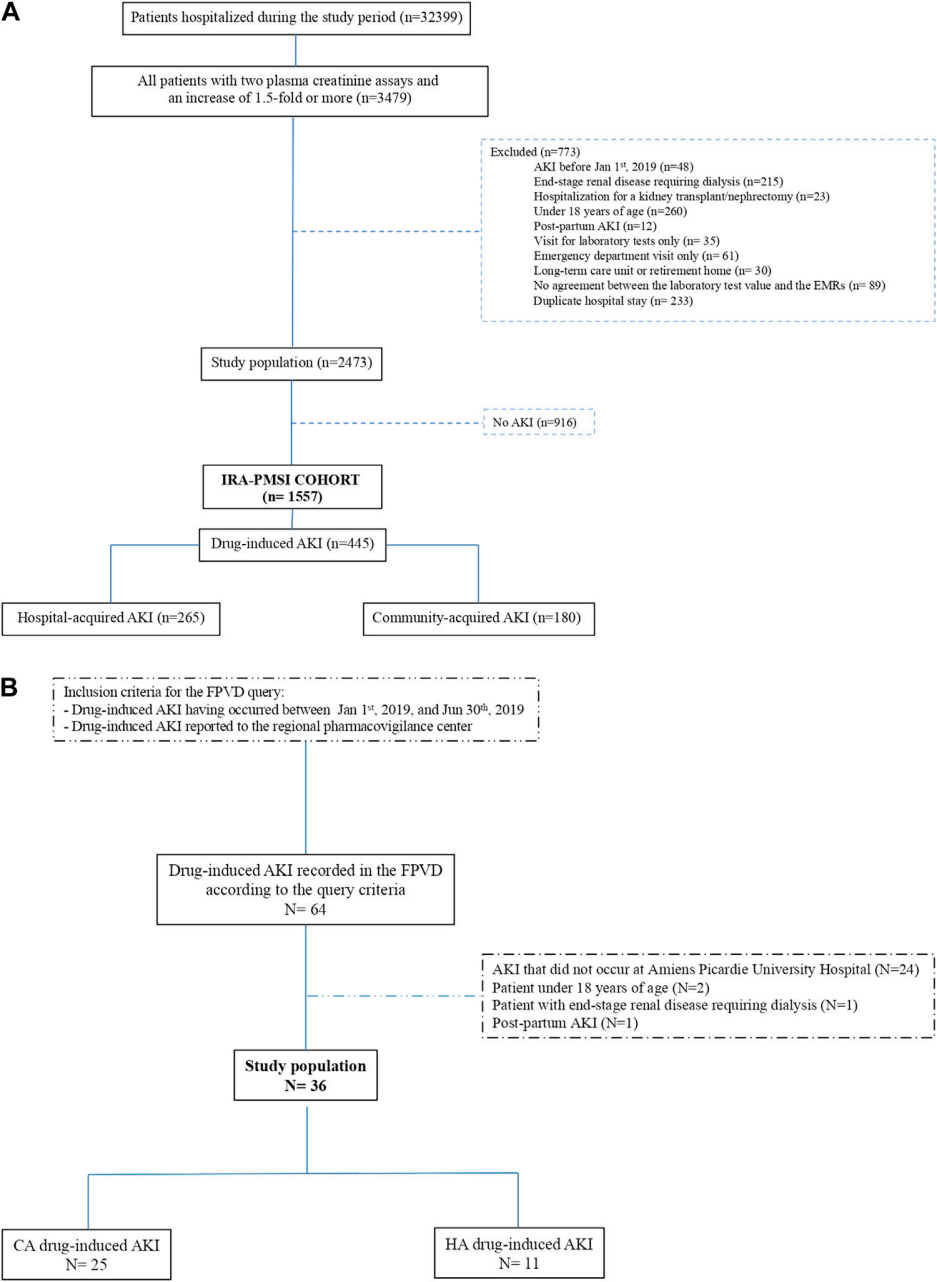
**(A)** Flow chart for the “IRA-PMSI” cohort, with the selection of drug-induced AKIs only (Rey et al., 2021). AKI, acute kidney injury; EMR, electronic medical record; PMSI, Programme de Médicalisation des Systèmes d'Information (the French national hospital discharge database). **(B)** Flow chart for the FPVD query. AKI, acute kidney injury; CA, community-acquired; HA, hospital-acquired; FPVD, French national pharmacovigilance database.

Our analysis of the cohort found that 445 of the 1,557 AKIs were drug-induced AKIs (Rey et al., 2021). CA-AKI accounted for 180 (40.4%) of the drug-induced AKIs, and HA-AKI accounted for 265 (59.6%) (Figure 2.A).

#### 3.1.2 French National Pharmacovigilance Database Query

The FPVD query detected 36 cases of drug-induced AKI over the same period occurred at Amiens Picardie University Hospital (i.e., from 1 January 2019, to 30 June 2019). We found that 25 (69.4%) of the drug-induced AKIs reported in the FPVD were CA-AKIs, and 11 (30.6%) were HA-AKIs (Figure 2B).

### 3.2 Description of all Cases of Drug-Induced Acute Kidney Injury Identified With Both the “IRA-PMSI” Cohort and the French National Pharmacovigilance Database

After the identification of cases listed in both data sources (i.e., 27 cases), we detected a total of 454 drug-induced AKIs (Figure 1). The patients’ characteristics are summarized in Table 1. The mean (standard deviation (SD)) age was 72.2 (14.6), and 50.5% of the patients were female. The most frequently observed comorbidities were hypertension (75.8%), a history of cardiovascular disease (CVD) (55.1%), and chronic kidney disease (CKD) (40.2%). Relative to patients with drug-induced AKI only found in the cohort (i.e. drug-induced AKIs not reported in the FPVD), patients with a drug-induced AKI reported in the FPVD were significantly less likely to have a history of CVD (57.8% vs. 30.6% respectively). These two groups of patents did not differ significantly with regard to the other demographic and clinical characteristics.

**TABLE 1:** Characteristics of the patients with drug-induced AKI, by data source.

	All drug-induced AKIs	Drug-induced AKIs reported in the FPVD^d^	Drug-induced AKIs recorded only in the “IRA-PMSI” cohort	*p* value
	*n*= 454	*n* = 36	*n*= 418	
Demographic variables				
Age, y, mean (SD)	72.2 (14.6)	70.3 (14.7)	72.3 (14.6)	0.43^a^
Women, *n* (%)	230 (50.5)	20 (55.6)	210 (50.1)	0.53^b^
BMI, kg/m^2^, mean (SD)	28.8 (6.9)	28.0 (6.6)	28.8 (6.9)	0.48^a^
Obese (BMI ≥30 kg/m^2^), *n* (%)	177 (38.9)	9 (25.0)	168 (40.1)	0.07^b^
Comorbidities^e^				
CKD, *n* (%)	183 (40.2)	13 (36.1)	170 (40.6)	0.60^b^
Hypertension, *n* (%)	345 (75.8)	25 (69.4)	320 (76.4)	0.35^b^
Diabetes, *n* (%)	179 (39.3)	15 (41.7)	164 (39.1)	0.77^b^
Dyslipidaemia	196 (43.1)	14 (38.9)	182 (43.4)	0.60^b^
History of CVD, *n* (%)	**253 (55.1)**	**11 (30.6)**	**242 (57.8)**	**0.002** ^b^
Cancer, *n(%)*	141 (31.0)	8 (22.2)	133 (31.8)	0.24^b^
History of AKI, *n(%)*	93 (20.4)	4 (11.1)	89 (21.2)	0.15^b^
History of ADRs, *n(%)*	157 (34.5)	10 (27.8)	147 (35.1)	0.47^c^

a Student’s *t* test.

b chi-squared test.

c Fisher’s exact test for patients with drug-induced AKI reported in the FPVD vs. patients with drug-induced AKI not reported in the FPVD.

d 27 cases were common to the two sources.

e according to the patients’ EMRs

Statistically significant differences are shown in bold type.

AKI, acute kidney injury; ADR, adverse drug reaction; BMI, body mass index; CKD, chronic kidney disease; CVD, cardiovascular disease; FPVD, french national pharmacovigilance database.

The “history of ADRs” item included ADRs that occurred before the index AKI and were mentioned in the patient’s EMRs.

The characteristics of the AKIs are summarized in Table 2. Compared with drug-induced AKIs identified in the cohort, the drug-induced AKIs reported in the FPVD were significantly more severe (respectively 17.7% and 52.8% were KDIGO grade 3) and were more likely to have been treated with dialysis (6.9% vs. 27.8% of cases, respectively).

**TABLE 2:** Characteristics of the episodes of drug-induced AKI, by data source.

	All drug-induced AKIs	Drug-induced AKIs reported in the FPVD^d^	Drug-induced AKIs recorded only in the “IRA-pmsi” cohort	*p* value
	*n*= 454	*n* = 36	*n*= 418	
About the AKI episode^e^				
Hospital acquired	**268 (58.9)**	**11 (30.6)**	**257 (61.3)**	**<0.001** ^a^
Number of drugs involved, *mean (SD)*	**2.4 (2.1)**	**5.6 (3.8)**	**2.2 (1.6)**	**<0.001** ^b^
Number of drugs involved, *n (%)*				**<0.001** ^c^
1 drug	186 (41.1)	4 (11.1)	182 (43.7)	
2 drugs	119 (26.2)	5 (13.9)	114 (27.2)	
More than 2 drugs	149 (32.7)	27 (75.0)	122 (29.1)	
KDIGO grade				**<0.001** ^a^
1 *(n,%)*	262 (57.8)	9 (25.0)	253 (60.6)	
2 (*n*,*%*)	99 (21.8)	8 (22.2)	91 (21.7)	
3 (*n*,*%*)	93 (20.4)	19 (52.8)	74 (17.7)	
Medical unit				**<0.001** ^a^
Nephrology, *n*(*%*)	68 (14.9)	13 (36.1)	55 (13.1)	
ICU, *n(%)*	63 (13.8)	12 (33.3)	51 (12.2)	
Other, *n(%)*	323 (71.3)	11 (30.6)	312 (74.7)	
Length of hospital stay, *med* (*IQR*)	11 (6.0–20.0)	11 (5.0–15.3)	11 (6.0–20.0)	0.87^b^
Length of hospital stay, *n (%)*				0.55^c^
Short (<7 days)	127 (27.9)	13 (36.1)	114 (27.2)	
Medium (7–29 days)	269 (59.4)	19 (52.8)	250 (59.9)	
Long (>29 days)	58 (12.7)	4 (11.1)	54 (12.9)	
Use of dialysis, *n(%)*	**39 (8.6)**	**10 (27.8)**	**29 (6.9)**	**<0.001** ^a^
Outcome				**<0.001** ^c^
Favourable, *n (%)*	316 (69.7)	24 (66.7)	292 (69.9)	
Onset or progression of CKD, *n (%)*	97 (21.3)	8 (22.2)	89 (21.2)	
Death, n (%)	36 (7.9)	2 (5.6)	34 (8.1)	
Unknown, *n (%)*	5 (1.1)	2 (5.6)	3 (0.7)	

a Chi-squared test.

b Student’s t-test.

c Fisher’s exact test for patients with drug-induced AKI reported in the FPVD vs. patients with drug-induced AKI not reported in the FPVD.

d according to the patients’ EMRs.

e 27 cases were common to the two sources.

AKI, acute kidney injury; CKD, chronic kidney disease; FPVD, french national pharmacovigilance database; KDIGO, Kidney Disease, Improving Global Outcomes; ICU, intensive care unit; IQR, interquartile range; SD, standard deviation.

Statistically significant differences are shown in bold type.

**FIGURE 3:**
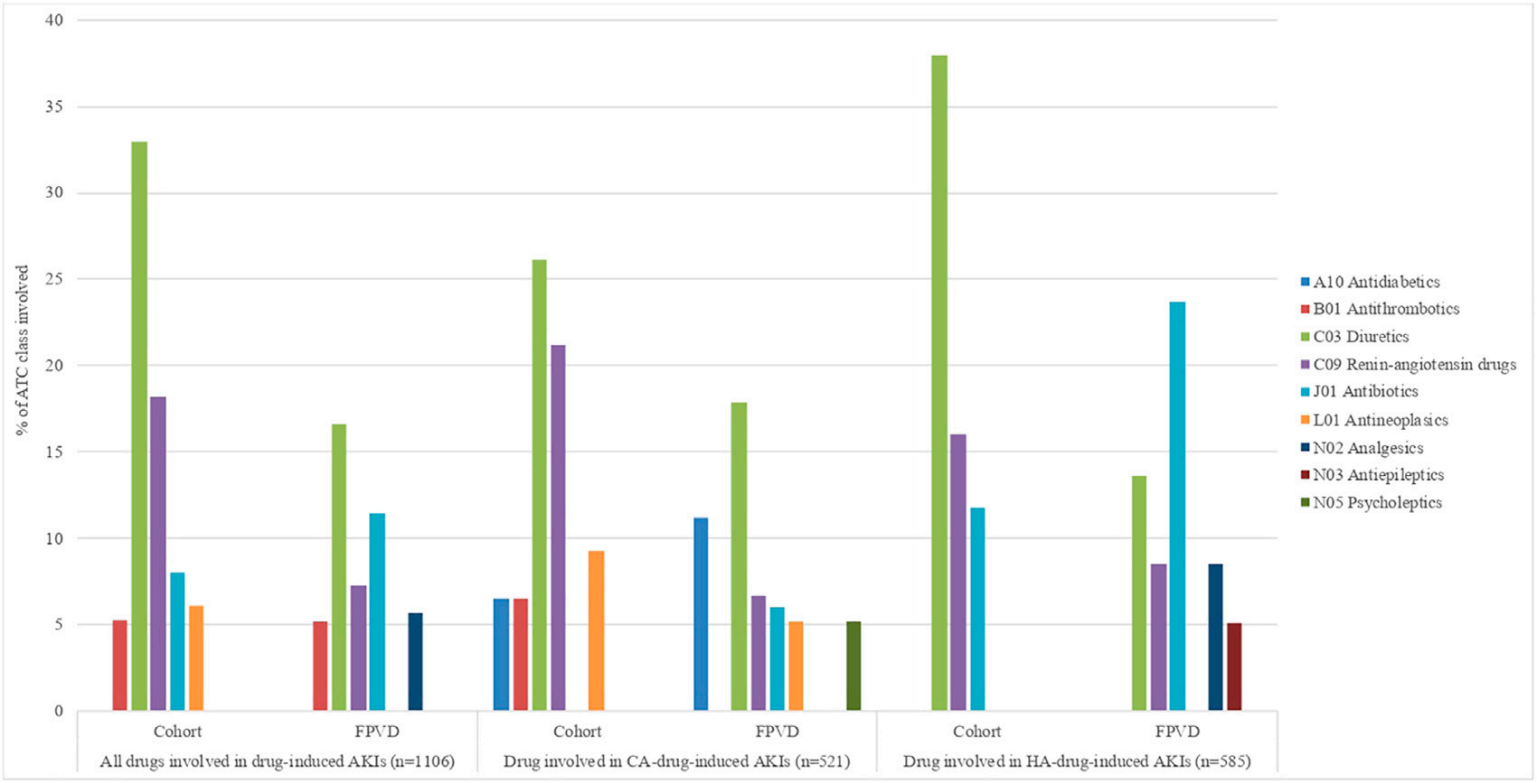
Drug-induced AKIs, according to the ATC classification level 2 drugs involved in more than 5% of cases. AKI, acute kidney injury; CA, community-acquired; FPVD, French national pharmacovigilance database; HA, hospital-acquired.

We found that a total of 1,106 drugs were involved in drug-induced AKIs. The main ATC classes involved were diuretics (30.1%), renin-angiotensin system drugs (16.3%), and antibiotics (8.6%) with sulfamethoxazole-trimethoprim as the main antibiotic involved (Figure 3). The main suspected drugs were furosemide (20.1%), spironolactone (5.5%), and ramipril (4.7%) (Supplementary Table S2).

The two ATC classes most frequently notified in the FPVD were antibiotics (proportion: 23.2%) and antithrombotics (proportion: 17.2%), whereas only 7.8% of the AKIs involving renin-angiotensin system drugs were reported in the FPVD (Supplementary Table S2).

### 3.3 Comparison of Hospital Acquired-Acute Kidney Injuries and Community-Acquired-Acute Kidney Injuries

Of the 454 drug-induced AKIs, 186 (41.0%) were classified as CA-AKIs and 268 (59.0%) were classified as HA-AKIs. The patients’ characteristics are summarized in Supplementary Table S3 for HA-AKIs and in Supplementary Table S4 for CA-AKIs. The characteristics of the HA-AKIs and CA-AKIs are summarized in Tables 3, 4, respectively.

**TABLE 3:** Characteristics of the episodes of hospital-acquired drug-induced AKI, by data source.

	All drug-induced AKIs	Drug-induced AKIs reported in the FPVD^c^	Drug-induced AKIs recorded only in the “IRA-pmsi” cohort	*p* value
	*n*= 268	*n* = 11	*n*= 257	
About the AKI episode^d^				
Number of drugs involved, *mean (SD)*	**2.2 (1.9)**	**5.6 (4.0)**	**2.1 (1.7)**	**0.01** ^a^
Number of drugs involved, *n (%)*				**0.01** ^b^
1 drug	126 (47.0)	2 (18.2)	124 (48.2)	
2 drugs	77 (28.7)	2 (18.2)	75 (29.2)	
More than 2 drugs	65 (24.3)	7 (63.6)	58 (22.6)	
KDIGO grade				**0.03** ^b^
1 (*n,%*)	184 (68.6)	4 (36.4)	180 (70.0)	
2 (*n,%*)	49 (18.3)	3 (27.3)	46 (17.9)	
3 (*n,%*)	35 (13.1)	4 (36.4)	31 (12.1)	
Medical unit				**<0.001** ^b^
Nephrology, *n*(*%*)	16 (6.0)	3 (27.3)	13 (5.1)	
ICU, *n*(*%*)	32 (11.9)	5 (45.5)	27 (10.5)	
Other, *n*(*%*)	220 (82.1)	3 (27.3)	217 (84.4)	
Length of hospital stay, *med* (*IQR*)	14.5 (8.0–26.0)	15.0 (11.5–22.5)	14.0 (8.0–26.0)	0.69^a^
Length of hospital stay, *n* (*%*)				0.67^b^
Short (<7 days)	45 (16.8)	1 (9.1)	44 (17.1)	
Medium (7–29 days]	173 (64.6)	9 (81.8)	164 (63.8)	
Long (>29 days)	50 (18.7)	1 (9.1)	49 (19.1)	
Use of dialysis, *n(%)*	13 (4.9)	2 (18.2)	11 (4.3)	0.09^b^
Outcome				0.60^b^
Favourable, *n (%)*	184 (68.7)	7 (63.6)	177 (68.9)	
Onset or progression of CKD, *n (%)*	54 (20.1)	2 (18.2)	52 (20.2)	
Death, n (%)	27 (10.1)	2 (18.2)	25 (9.7)	
Unknown, *n (%)*	3 (1.1)	0 (0)	3 (1.2)	

a Student’s *t*-test or chi-squared test.

b Fisher’s exact test for patients with drug-induced AKI reported in the FPVD vs. patients with drug-induced AKI not reported in the FPVD.

c 8 cases were common to the two sources.

d according to the patients’ EMRs.

AKI, acute kidney injury; CKD, chronic kidney disease; FPVD, french national pharmacovigilance database; KDIGO, Kidney Disease, Improving Global Outcomes; ICU, intensive care unit; IQR, interquartile range; SD, standard deviation.

Statistically significant differences are shown in bold type.

**TABLE 4:** Characteristics of the episodes of community-acquired drug-induced AKI, by data source.

	All drug-induced AKIs	Drug-induced AKIs reported in the FPVD^d^	Drug-induced AKIs recorded only in the “IRA-pmsi” cohort	*p* value
	*n*= 186	*n* = 25	*n*= 161	
About the AKI episode^e^				
Number of drugs involved, *mean (SD)*	**2.8 (2.2)**	**5.5 (3.8)**	**2.3 (1.4)**	**<0.001** ^b^
Number of drugs involved, *n (%)*				**<0.001** ^b^
1 drug	61 (32.6)	2 (8.0)	59 (36.4)	
2 drugs	66 (22.5)	3 (12.0)	63 (24.1)	
More than 2 drugs	59 (44.9)	20 (80.0)	39 (39.5)	
KDIGO grade				**0.003** ^b^
1 *(n,%)*	78 (42.2)	5 (20.0)	73 (45.7)	
2 *(n,%)*	50 (26.7)	6 (20.0)	45 (27.8)	
3 *(n,%)*	58 (31.0)	15 (60.0)	43 (26.5)	
Medical unit				**0.03** ^c^
Nephrology, *n(%)*	52 (27.8)	10 (40.0)	42 (25.9)	
ICU, *n(%)*	31 (16.6)	7 (28.0)	24 (14.8)	
Other, *n(%)*	103 (55.6)	8 (32.0)	96 (59.3)	
Length of hospital stay, *med [IQR]*	8.0 [4.0–13.0]	7.0 [5.0–13]	8.0 [4.0–12.8]	0.36^b^
Length of hospital stay, *n (%)*				0.09^b^
Short (<7 days)	82 (43.9)	12 (48.0)	70 (43.2)	
Medium (7–29 days]	96 (51.9)	10 (40.0)	86 (53.7)	
Long (>29 days)	8 (4.3)	3 (12.0)	5 (3.1)	
Use of dialysis, *n(%)*	**26 (13.9)**	**8 (32.0)**	**18 (11.1)**	**0.005** ^c^
Outcome				**0.03** ^b^
Favourable, *n (%)*	132 (71.1)	17 (68.0)	115 (71.6)	
Onset or progression of CKD, *n (%)*	43 (23.0)	6 (24.0)	37 (22.8)	
Death, n (%)	9 (4.8)	0 (0)	9 (5.6)	
Unknown, *n (%)*	2 (1.1)	2 (8.0)	0 (0)	

a Student’s *t* test or chi-squared test.

b Fisher’s exact test.

c Chi-squared test for patients with drug-induced AKI reported in the FPVD vs. patients with drug-induced AKI not reported in the FPVD.

d 19 cases were found in both data sources.

e according to the patients’ EMRs.

Statistically significant differences are shown in bold type.

AKI, acute kidney injury; CKD, chronic kidney disease; FPVD, French national pharmacovigilance database; KDIGO, Kidney Disease, Improving Global Outcomes; ICU, intensive care unit; IQR, interquartile range; SD standard deviation.

We found that respectively 585 and 521 different drugs were involved in HA-AKIs and CA-AKIs. After diuretics and renin-angiotensin drugs, the most frequently involved drug classes were antibiotics in the HA-AKI subgroup (13.0%), when the main suspected drugs were furosemide in diuretics class, ramipril in renin-angiotensin drugs class and sulfamethoxazole-trimethoprim in antibiotics class (Figure 3 and Supplementary Table S5). In the CA-AKI subgroup, diuretics and renin-angiotensin drugs still the most frequently involved drug classes followed by antineoplasics (cisplatin as the main drug involved), antidiabetics (metformin as the main drug involved) and antithrombotics (apixaban as the main drug involved) (Figure 3 and Supplementary Table S6).

When considering the ATC classes involved in more than 5% of drug-induced AKIs, antibiotics (sulfamethoxazole-trimethoprim as the main drug involved) was the most reported ATC classes in the FPVD (18.4%) in the HA-AKI subgroup (Supplementary Table S5), whereas it was antidiabetics (metformine as the main drug involved) in CA-AKI group (37.5%) (Supplementary Table S6).

### 3.4 Estimation of the Prevalence of Drug-Induced Acute Kidney Injury

According to the capture-recapture method, the estimated total number of drug-induced AKIs was 593 [95%CI: 485–702]. We detected 454 drug-induced AKIs out of an estimated total of 593 and so considered that 139 drug-induced AKIs had not been detected (Table 5).

**TABLE 5:** Estimation of the number of drug-induced AKIs using the capture-recapture method, as a function of the place of acquisition.

	Number of observed drug-induced AKIs			Estimate of the total number of drug-induced AKIs		
	Cohort	FPVD	Matches	X	N	95%CI
*Non-stratified analysis*						
All drug-induced AKIs	445	36	27	139	593	[485–702]
*Analysis by place acquisition*						
Community-acquired	180	25	19	51	237	[188–286]
Hospital-acquired	265	11	8	96	364	[235–494]

AC, community-acquired; HA, hospital-acquired; FPVD, french national pharmacovigilance database; CI, confidence interval; AKI, acute kidney injury; N, total number of drug-induced AKIs; X, number of non-identified drug-induced AKIs.

We identified 265 drug-induced HA-AKIs and 180 drug-induced CA-AKIs in the hospital cohort; respectively 11 and 25 of in the FPVD. Eight HA-AKIs and 19 CA-AKIs were common to the two data sources. According to the capture-recapture method, the estimated total numbers [95%CI] of drug-induced HA-AKIs and CA-AKIs were respectively 364 [235–494] and 237 [188–286]. The estimated number of non-identified cases was 96 for drug-induced HA-AKIs and 51 for drug-induced CA-AKIs (Table 5).

**FIGURE 4:**
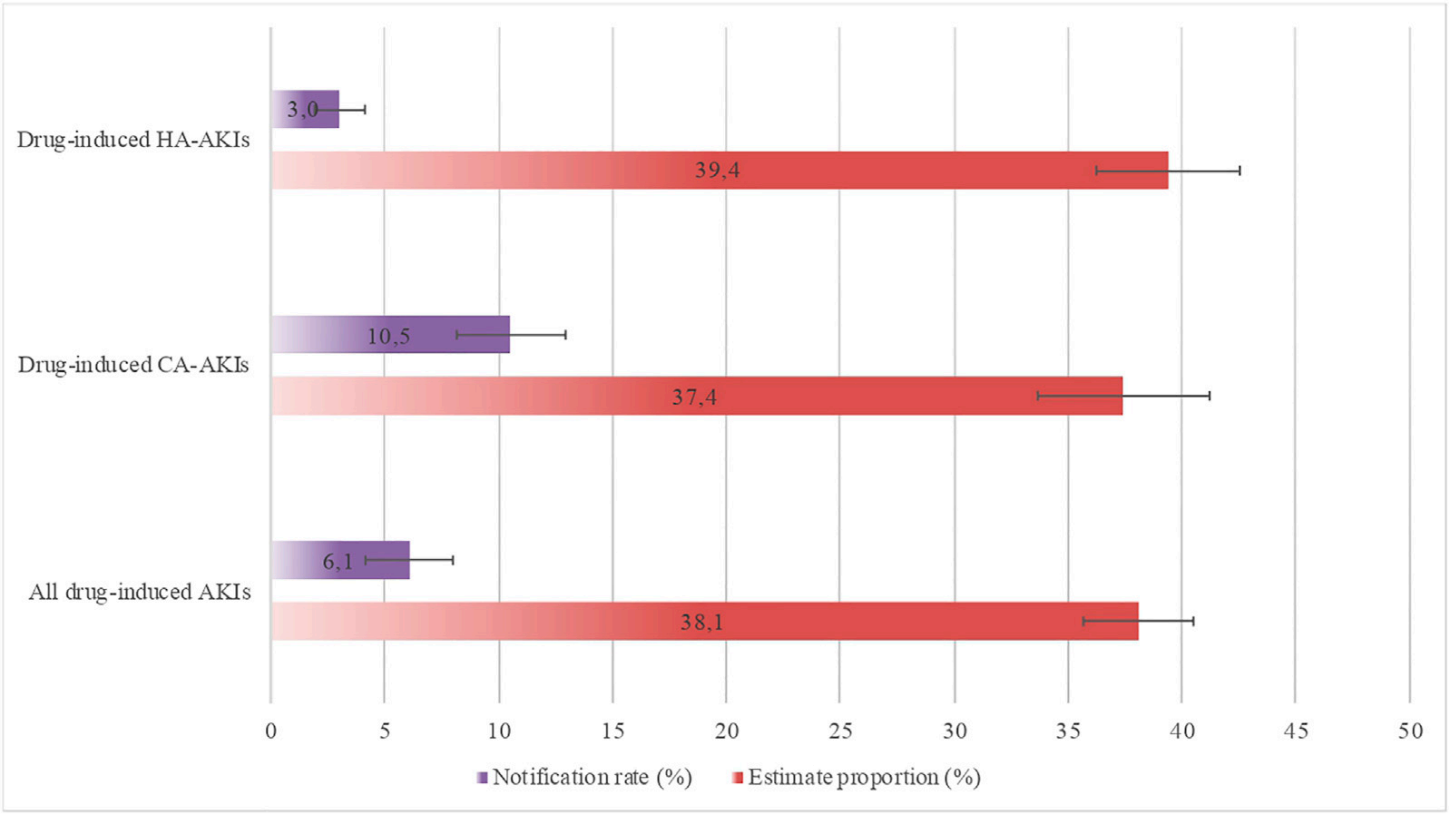
The FPVD notification rates and estimated proportions for all drug-induced AKIs, as a function of the place of acquisition. AKI, acute kidney injury; CA, community-acquired; FPVD, French national pharmacovigilance database; HA, hospital acquired.

When considering all 1,557 AKIs, the estimated prevalence [95%CI] of all drug-induced AKIs during a hospital stay was 38.1% [35.67–40.50]. The FPVD notification rate was 6.1% [4.15–7.99]. The estimated prevalence of drug-induced HA-AKIs was 39.4% [36.24–42.54], when considering all 924 cases. The estimated prevalence of drug-induced CA-AKI was 37.4% [33.67–41.21], when considering all 633 cases (Rey et al., 2021). The FPVD notification rate was 3.0% [1.92–4.13] for HA-AKIs and 10.5% [8.16–12.94] for CA-AKIs (Figure 4).

**FIGURE 5:**
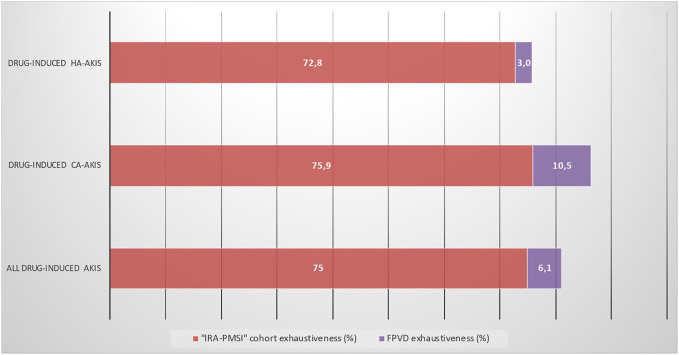
Exhaustiveness of the “IRA-PMSI” cohort and the FPVD query for identifying all drug-induced AKIs, as a function of where the place of acquisition. AKI, acute kidney injury; CA-AKI, community-acquired acute kidney injury; HA-AKI, hospital-acquired acute kidney injury; FPVD, French national pharmacovigilance database.

The exhaustiveness of detection of drug-induced AKI in our cohort was 75% (75.9% for CA-AKI and 72.8% for HA-AKI) (Figure 5).

## 4 Discussion

### 4.1 Key Points

The capture-recapture method is a useful tool for estimating frequencies when several sources of information are available and can be matched. Application of this method to hospital and pharmacovigilance databases enabled us to estimate the prevalence of drug-induced AKI among hospitalized patients with AKI: 38.1% [35.7–40.5]. The most frequently suspected drug classes were diuretics (30.1%), renin-angiotensin system drugs (16.3%), and antibiotics (8.6%). Drug-induced AKIs reported in the FPVD were more likely to be severe and to require haemodialysis than cases of drug-induced AKI identified in the PMSI hospital discharge database.

The prevalence of drug-induced AKI in the present study was higher than the values reported in the literature (Wu and Huang, 2018; Robert et al., 2019; Rolland et al., 2021). This disparity might be due to the use of different methods to identify AKIs. Indeed, most of the literature studies used International Statistical Classification of Diseases and Related Health Problems, 10^th^ Revision (ICD-10) codes, whereas we screened EMRs and meticulously applied our algorithm to each AKI episode. Our method probably enabled us to identify a higher proportion of cases in general and CA-AKIs in particular. Using the same capture-recapture method, Rolland et al. found that 20.0% of AKIs were drug-induced AKI (Rolland et al., 2021). However, cases from the hospital database were identify by using AKI ICD-10 codes as the main diagnosis or an associated diagnosis—leading to underestimation of the number of cases (relative to the identification process applied in the present study (Rey et al., 2021)).

Our results show that drug-induced AKI is a common and probably underestimated condition in both community and hospital settings. Given the multifactorial origin of AKI, it is important to identify avoidable factors in order to facilitate prevention and management. Drug-induced AKIs are often avoidable and so are potentially the easiest aetiologies to manage: Laville et al.’s prospective, multicentre study of the CKD-REIN cohort showed that 40% of drug-induced AKIs due to renin-angiotensin system drugs and 33% of drug-induced AKIs due to diuretics were avoidable or potentially avoidable (Olivier et al., 2005). By focusing on community-acquired AKI, Robert et al. found that 7.1% of drug-induced AKIs were preventable and 59.7% were potentially preventable (Robert et al., 2019). Our results emphasize the significance of AKIs in both areas and suggest that the management of CA-AKIs and HA-AKIs should be personalized in order to accelerate the patient’s recovery. Indeed, as we initially hypothesized, we have shown that CA and HA-AKIs present differences in term of drugs involved probably in relation with differences of drugs use and AKI causes.

### 4.2 Notification Rate in the French National Pharmacovigilance Database

Our results highlight the lack of exhaustiveness of pharmacovigilance databases in identifying drug-induced AKI; indeed, only 6.1% of the drug-induced AKIs in the cohort were recorded in the FPVD. The FPVD notification rate differed significantly as a function of the origin, with 10.5% for CA-AKIs and only 3.0% for HA-AKIs. Drug-induced AKI seems to be more often reported in the FPVD if it is the main diagnosis of hospitalization. Greater awareness among health professionals may be needed when dealing with drug-induced HA-AKI. However, the pharmacovigilance database was designed to identify safety signals, rather than to be exhaustive. In contrast, the exhaustiveness for the detection of drug-induced AKI in the cohort constituted with chart review and PMSI queries was 75%. Previous comparisons of hospital and pharmacovigilance databases have given similar results: more ADRs were identified by analyzing hospital databases than by spontaneous reporting (Lugardon et al., 2006). In fact, we found that characteristic of the AKIs influenced their reporting. Drug-induced AKIs were more likely to be reported to the FPVD if they had been severe (52.8% of KDIGO grade 3 AKIs vs. 17.7% of KDIGO grade 1 or 2 AKIs) and if dialysis had been required. This is in line with literature data: in an observational descriptive study using the FPVD, Moulis et al., have shown that practitioners report more frequently severe ADRs than non-severe ADRs, especially after the first years of marketing (Moulis et al., 2012).

### 4.3 Drugs Involved in Community Acquired- and Hospital Acquired-Acute Kidney Injuries

The main drugs involved in AKIs in the present study were similar to those mentioned in the literature (Pierson-Marchandise et al., 2017; Rolland et al., 2021). The drug involved in CA-AKIs were not exactly the same as those involved in reported HA-AKIs. Antibiotics were frequently involved in HA-AKIs and constituted the most frequently reported ATC class in the FVPD, which is in line with the literature data (Liu et al., 2020). Antibiotics can induce AKI through several mechanisms (Ghane Shahrbaf and Assadi, 2015). Indeed, trimethoprim/sulfamethoxazole can led to AKI by inducing acute interstitial nephritis or acute tubular necrosis, or by forming crystals in the urine of volume-depleted patients (Fraser et al., 2012).

Antineoplastic and antithrombotic drugs were frequently involved in CA-AKIs. This might have been due to the characteristics of the population in the community, such as a greater comorbidity burden and more frequent polypharmacy for chronic health conditions. Antineoplastic agents such as cisplatin can produce chronic interstitial injury by inducing oxidative stress, apoptosis, necrosis, local and systemic inflammation, the release of inflammatory mediators, and autophagy (Holditch et al., 2019). A decline in renal function is common among patients treated with oral anticoagulants. In a retrospective study of a large administrative database in the United States, Yao et al., showed that 1 in 7 patients had experienced an episode of AKI at some time during a 2-year period (Yao et al., 2017). In the present study, apixaban was the leading drug in the ATC oral anticoagulant class. It has been shown that apixaban can induce severe acute tubular necrosis (Brodsky et al., 2017). In addition, direct oral anticoagulant are relatively recent class that could lead to more frequently reports corresponding to a reporting bias. The high observed prevalence of CA-AKI and HA-AKI and the differences in the drug classes involved in the CA-AKI and HA-AKI subgroups highlight the need to adjust and improve the management of these injuries as a function of the affected population.

### 4.4 Strengths and Limits

Our study had several strengths. Firstly, we used the capture-recapture methodology, which is known to be effective in estimating the proportion of ADRs by bringing together several sources of incomplete information. This method allowed us to estimate the proportion of drug-induced AKIs as precisely as possible. The assessment of a specific cohort of patients with drug-induced AKI might be a valuable tool for increasing the detection rate. In the present study, the proportion of identified drug-induced AKI was 75% in our cohort but only 6.1% in the FPVD. The assessment of our cohort significantly increased the number of drug-induced AKIs detected, relative to spontaneous notifications. Secondly, we used the Naranjo ADR probability scale to identify cases of drug-induced AKI. This simple, validated questionnaire can be answered quickly (Naranjo et al., 1981). A rapid method is needed because the incidence of adverse events can be estimated only from cases identified as definite or probable ADRs. Administrative databases are an important source of information and could be very useful for detecting drug-induced AKI. Analysis of a combination of the two data sources with the capture-recapture method enables one to calculating the proportion of drug-induced AKIs in particular populations, such as those with CA-AKI and HA-AKI. Since drug-induced AKIs account for a large (and possible underestimated) proportion of CA- and HA-AKIs and are mostly avoidable, our present findings might help to improve awareness and management of drug-induced AKIs, limit the occurrence of these events, and thus reduce kidney damage.

Our study had some limitations. Since we conducted a single-centre study in a university hospital in northern France, our findings might reflect the prescribing habits of our local general practitioners and the hospital’s clinicians. However, on the basis of the literature data, we expect the clinical characteristics of patients with drug-induced AKI to be much the same in France as in other countries (Rey et al., 2021). Also, the independence of our sources could not be statistically confirmed in a log-linear model because there were only two (Gallay et al., 2002; Lugardon et al., 2006). However, the independence could be judged qualitatively because the cohort was constituted via information collected by our hospital’s medical information department, which does not usually report ADRs to the regional pharmacovigilance centre (Rolland et al., 2021). A common limitation of cohort based on diagnosis codes is the consistency of the coding. But AKI represents an event that induces additional costs for hospitals and so prompts them to code it (Chertow et al., 2005). Also, we previously demonstrated using a set of ICD 10 codes for AKI that the codes found corresponded to those mostly used to code AKIs (Rey et al., 2021). Finally, we did not have information about length of treatment and suspected drugs’rechallenge. We could not study whether they were risk factors for drug-induced AKI, nor their influence on renal recovery.

## 5 Conclusion

Application of the capture-recapture method to two incomplete data sources can improve the ability to identify and quantify ADRs and, as we showed, drug-induced AKIs—particularly in a hospital setting, where under-reporting is a particular problem. The construction of specific ADR cohorts might enhance the reporting of ADRs (as observed for drug-induced AKIs) and thus improve drug safety monitoring. Linking the PMSI administrative database to specific cohorts (such as our cohort, with EMR analysis) might further increase the detection of ADRs (and not just drug-induced AKIs); studying such an approach would be a logical next step such as a patient follow-up after hospital discharge to study the effect of a possible drug rechallenge.

## Data Availability

The datasets presented in this article are not readily available because due to french law, dataset cannot be shared. Requests to access the datasets should be directed to Liabeuf.Sophie@chu-amiens.fr
