# Supplementary material for: Use of the Capture-Recapture Method to Estimate the Frequency of Community- and Hospital-Acquired Drug-Induced Acute Kidney Injuries in French Databases
Source: Front Pharmacol. 2022 Jul 5;13:899164. doi: 10.3389/fphar.2022.899164 (PMC9294528; doi:10.3389/fphar.2022.899164)
Supplement: Supplementary file 1 [file DataSheet1.docx]

Supplementary Material

# Supplementary data

## Supplementary method

The *Programme de Médicalisation des Systèmes d'Information* (PMSI) is the French national hospital discharge summary database. It includes the hospital identifier, hospital stay start and end dates, and the diagnostic codes (a primary diagnosis, a related diagnosis, and up to 30 associated diagnoses, coded according to the International Classification of Diseases, 10th Revision (ICD-10)) (Boudemaghe & Belhadj, 2017). At Amiens Picardie University Hospital (Amiens, France), we conducted a single-centre retrospective study (“IRA-PMSI”) from January 1^st^, 2019, to June 30^th^, 2019, in order to build a cohort of hospitalized patients suffering from AKIs. The main inclusion criteria were age over 18 and at least two available plasma creatinine measurements with a 1.5-fold increase or more during the hospital stay. This data extraction was possible thanks to a collaboration with the medical information department. The exclusion criteria included a diagnosis of end-stage renal disease requiring dialysis, kidney transplantation during the study period, postpartum AKI, hospitalization in an emergency department or a long-term care unit only, and a hospital visit for laboratory tests only. A single AKI per hospital stay was studied. If a patient had several AKIs in a single hospital stay, only the first AKI was selected for analysis. If a patient had several hospital stays with several episodes of AKI, we considered each episode of AKI independently. Cases of AKIs were defined as follows: we first looked manually for a physician-diagnosed AKI in the patient’s EMRs and then for a plasma creatinine level elevated to over 1.5 times baseline, according to the KDIGO creatinine criteria (**Supplementary Figure 1**). The diagnosis of an AKI was confirmed if one or the other suggested an AKI event.

Lastly, the investigating pharmacologists distinguished between CA-AKI and HA-AKI, according to the availability of a baseline creatinine measurement, the physician’s diagnosis in the EMRs, and whether or not AKI was the condition leading to admission (for more details, see **Supplementary Figure 1**) (Rey et al., 2021). This approach ensured that we would not miss an AKI episode when a baseline creatinine value was missing (after measurement in a clinical laboratory outside hospital, for example), and is known to be highly specific for AKI.

## References

Boudemaghe, T., & Belhadj, I. (2017). Data Resource Profile : The French National Uniform Hospital Discharge Data Set Database (PMSI). *International Journal of Epidemiology*, *46*(2), 392‑392d. https://doi.org/10.1093/ije/dyw359

Rey, A., Gras-Champel, V., Balcaen, T., Choukroun, G., Masmoudi, K., & Liabeuf, S. (2021). Use of a hospital administrative database to identify and characterize community-acquired, hospital-acquired and drug-induced acute kidney injury. *Journal of Nephrology*. supplementaryhttps://doi.org/10.1007/s40620-021-01174-z

## Supplementary figure

**Supplementary Figure 1: Algorithm for identifying cases of CA-AKI or HA-AKI**

CKD: chronic kidney disease; AKI: acute kidney injury; CA-AKI: community-acquired acute kidney injury; HA-AKI: hospital-acquired acute kidney injury; EMR: electronic medical record

# Supplementary Tables

Supplementary Table 1. MedDRA terms included in the standardized MedDRA query for “acute renal failure”

| **MedDRA terms** | |
| --- | --- |
| Acute kidney injury | Hypercreatininaemia |
| Acute phosphate nephropathy | Hyponatriuria |
| Albuminuria | Intradialytic parenteral nutrition |
| Anuria | Kidney injury molecule-1 |
| Azotaemia | Neonatal anuria |
| Blood creatinine abnormal | Nephritis |
| Blood creatinine increased | Nephropathy toxic |
| Blood urea abnormal | Oedema due to renal disease |
| Blood urea increased | Oliguria |
| Blood urea nitrogen/creatinine ratio increased | Peritoneal dialysis |
| Continuous haemodiafiltration | Prerenal failure |
| Creatinine renal clearance abnormal | Protein urine present |
| Creatinine renal clearance decreased | Proteinuria |
| Creatinine urine abnormal | Renal failure |
| Creatinine urine decreased | Renal failure neonatal |
| Crystal nephropathy | Renal function test abnormal |
| Dialysis | Renal impairment |
| Foetal renal impairment (PT narrow) | Renal impairment neonatal |
| Fractional excretion of sodium | Renal transplant |
| Glomerular filtration rate abnormal | Renal tubular disorder |
| Glomerular filtration rate decreased | Renal tubular dysfunction |
| Haemodialysis | Renal tubular injury |
| Haemofiltration | Renal tubular necrosis |
| Tubulointerstitial nephritis | Urea renal clearance decreased |
| Urine output decreased |  |

Supplementary Table 2. Drug-induced acute kidney injuries, according to the ATC classification level 2 drugs involved in more than 5% of cases, and the 3 most frequent drugs for each class

| **ATC classes** | **Drugs** | **All suspected drugs** | **Drugs suspected in the cohort** | **Drugs suspected in the FPVD** | Proportion of AKIs recorded in the FPVD ***** |
| --- | --- | --- | --- | --- | --- |
|  |  | n=1106 | n=913 | n=193 |  |
| **C03** | **Diuretics** | **333 (30.1)** | **301 (33.0)** | **32 (16.6)** | **9.6** |
| C03CA01 | Furosemide | 222 (20.1) | 211 (23.1) | 11 (5.7) | 5.0 |
| C03DA01 | Spironolactone | 61 (5.5) | 52 (5.7) | 9 (4.7) | 14.8 |
| C03AA03 | Hydrochlorothiazide | 32 (2.9) | 25 (2.7) | 7 (3.6) | 21.9 |
| **C09** | **Renin-angiotensin system drugs** | **180 (16.3)** | **166 (18.2)** | **14 (7.3)** | **7.8** |
| C09AA05 | Ramipril | 52 (4.7) | 49 (5.4) | 3 (1.6) | 5.8 |
| C09AA04 | Perindopril | 32 (2.9) | 28 (3.1) | 4 (2.1) | 12.5 |
| C09CA06 | Candesartan | 29 (2.6) | 26 (2.9) | 3 (1.6) | 10.3 |
| **J01** | **Antibiotics** | **95 (8.6)** | **73 (8.0)** | **22 (11.4)** | **23.2** |
| J01EE01 | Sulfamethoxazole-trimethoprim | 15 (1.4) | 12 (1.3) | 3 (1.6) | 20.0 |
| J01CR02 | Amoxicillin-clavulanic acid | 9 (0.8) | 8 (0.9) | 1 (0.5) | - |
| J01GB03 | Gentamicin | 7 (0.6) | 7 (0.8) | 0 (0) | - |
| **L01** | **Antineoplastics** | **63 (5.7)** | **56 (6.1)** | **7 (3.6)** | **11.1** |
| L01XA01 | Cisplatin | 10 (0.9) | 10 (1.1) | 0 (0) | - |
| L01XA02 | Carboplatin | 7 (0.6) | 7 (0.8) | 0 (0) | - |
| L01BA01 | Methotrexate | 7 (0.6) | 6 (0.7) | 1 (0.5) | - |
| **B01** | **Antithrombotics** | **58 (5.2)** | **48 (5.3)** | **10 (5.2)** | **17.2** |
| B01AF02 | Apixaban | 11 (1.2) | 12 (1.2) | 2 (1.0) | 15.4 |
| B01AC06 | Acetylsalicylic acid | 9 (0.8) | 8 (0.9) | 2 (1.0) | - |
| B01AA12 | Fluindione | 10 (0.8) | 6 (0.7) | 3 (1.6) | 33.3 |

AKI, acute kidney injury; FPVD, French national pharmacovigilance database

* The proportion of recorded AKIs is given for drugs involved in more than 10 cases of drug-induced AKI

Supplementary Table 3. Characteristics of patients with drug-induced HA-AKI, by data source

|  | **All drug-induced AKIs** | **Drug-induced AKIs reported in the FPVD*** | **Drug-induced AKIs recorded only in the "IRA-PMSI" cohort** | ***P* value** |
| --- | --- | --- | --- | --- |
|  | **n= 268** | **n=11** | **n= 257** |  |
| **Demographic variables** |  |  |  |  |
| Age, y, mean (SD) | 74.6 (14.0) | 71.5 (18.0) | 74.8 (13.9) | 0.57^a^ |
| Women, *n* (%) | 138 (51.5) | 5 (45.5) | 133 (51.8) | 0.68^b^ |
| BMI, kg/m^2^, mean (SD) | 28.8 (6.8) | 31.4 (8.7) | 28.7 (6.8) | 0.32^a^ |
| Obese (BMI ≥30 kg/m^2^), *n* (%) | 110 (41.0) | 5 (45.5) | 105 (40.9) | 0.76^b^ |
| **Comorbidities ^£^** |  |  |  |  |
| CKD, *n* (%) | 98 (36.6) | 3 (27.3) | 95 (37.0) | 0.75^c^ |
| Hypertension, *n* (%) | 203 (75.7) | 7 (63.6) | 196 (76.3) | 0.34 ^b^ |
| Diabetes, *n* (%) | 99 (36.9) | 4 (36.4) | 95 (37.0) | 1^c^ |
| Dyslipidaemia | 125 (46.6) | 5 (45.5) | 120 (46.7) | 0.94 ^b^ |
| History of CVD, *n* (%) | 168 (62.7) | 4 (36.4) | 164 (63.8) | 0.11 ^c^ |
| Cancer, *n(%)* | 70 (26.1) | 2 (18.2) | 68 (26.5) | 0.73 ^c^ |
| **History of kidney transplant, *n(%)*** | **8 (3.0)** | **2 (18.2)** | **6 (2.3)** | **0.04 ^c^** |
| History of AKI, *n(%)* | 51 (19.0) | 1 (9.1) | 50 (19.5) | 0.70 ^c^ |
| History of ADRs, *n(%)* | 87 (32.5) | 4 (36.4) | 83 (32.3) | 0.75 ^c^ |

AKI, acute kidney injury; ADR, adverse drug reaction; BMI, body mass index; CKD, chronic kidney disease; CVD, cardiovascular disease; FPVD: French national pharmacovigilance database

^a^: Student’s t test; ^b^: Pearson’s chi-squared test; ^c^: Fisher’s exact test for patients with drug-induced AKI reported in the FPVD vs. patients with drug-induced AKI not reported in the FPVD.

The “history of ADRs” item included ADRs that occurred before the index AKI and were mentioned in the patient’s EMRs.

^£^ according to the patients’ EMRs.

Statistically significant differences are shown in bold type.

*8 cases were common to the two sources

Supplementary Table 4. Characteristics of patients with drug-induced CA-AKIs, by data source

|  | All drug-induced AKIs | Drug-induced AKIs reported in the FPVD* | Drug-induced AKIs recorded only in the "IRA-PMSI" cohort | ***P* value** |
| --- | --- | --- | --- | --- |
|  | n= 186 | n=25 | n= 161 |  |
| **Demographic variables** |  |  |  |  |
| Age, y, mean (SD) | 68.6 (14.7) | 69.8 (13.3) | 68.4 (15.0) | 0.64^a^ |
| Women, *n* (%) | 92 (49.2) | 15 (60.0) | 77 (47.5) | 0.25^b^ |
| **BMI, kg/m^2^, mean (SD)** | **28.7 (7.0)** | **26.5 (4.9)** | **29.1 (7.2)** | **0.03** ^a^ |
| **Obese (BMI ≥30 kg/m^2^), *n* (%)** | **67 (35.8)** | **4 (16.0)** | **63 (38.9)** | **0.03^c^** |
| **Comorbidities ^£^** |  |  |  |  |
| CKD, *n* (%) | 85 (45.5) | 10 (40.0) | 75 (46.3) | 0.67^b^ |
| Hypertension, *n* (%) | 142 (75.9) | 18 (72.0) | 124 (76.5) | 0.62 ^b^ |
| Diabetes, *n* (%) | 80 (42.8) | 11 (44.0) | 69 (42.6) | 0.89 ^b^ |
| Dyslipidaemia | 71 (38.0) | 9 (36.0) | 62 (38.3) | 0.83 ^b^ |
| History of CVD, *n* (%) | 85 (45.5) | 7 (28.0) | 78 (48.1) | 0.06 ^b^ |
| Cancer, *n(%)* | 71 (38.0) | 6 (24.0) | 65 (40.1) | 0.12 ^b^ |
| History of AKI, *n(%)* | 42 (22.5) | 3 (12.0) | 39 (24.1) | 0.21^c^ |
| History of ADRs, *n(%)* | 70 (37.4) | 6 (24.0) | 64 (39.5) | 0.14^b^ |

AKI, acute kidney injury; ADR, adverse drug reaction; BMI, body mass index; CKD, chronic kidney disease; CVD, cardiovascular disease; FPVD: French national pharmacovigilance database

^a^: Student’s t test; ^b^: Pearson’s chi-squared test; ^c^: Fisher’s exact test for patients with drug-induced AKI reported in the FPVD vs. patients with drug-induced AKI not reported in the FPVD.

The “history of ADRs” item included ADRs that occurred before the index AKI and were mentioned in the patient’s EMRs.

*19 cases were common to the two sources.

£ according to the patients’ EMRs.

Statistically significant differences are shown in bold type.

Supplementary Table 5. Drug-induced HA-acquired AKIs, according to the ATC classification level 2 drugs involved in more than 5% of cases, and the 3 most frequently involved drugs in each class

| **ATC classes** | **Drugs** | **All suspected drugs** | **Drugs suspected in the cohort** | **Drugs suspected in the FPVD** | Proportion of AKIs recorded in the FPVD **(%)*** |
| --- | --- | --- | --- | --- | --- |
|  |  | **n=585** | **n=526** | **n=59** |  |
| **C03** | **Diuretics** | **208 (35.6)** | **200 (38.0)** | **8 (13.6)** | **3.8** |
| C03CA01 | Furosemide | 155 (26.5) | 151 (28.7) | 4 (5.1) | 2.6 |
| C03DA01 | Spironolactone | 33 (5.6) | 30 (5.7) | 3 (5.1) | 9.1 |
| C03AA03 | Hydrochlorothiazide | 14 (2.4) | 13 (2.5) | 1 (1.7) | 7.1 |
| **C09** | **Renin-angiotensin system drugs** | **89 (15.2)** | **84 (16.0)** | **5 (8.5)** | **5.6** |
| C09AA05 | Ramipril | 31 (5.3) | 30 (5.7) | 1 (1.7) | 3.2 |
| C09AA04 | Perindopril | 17 (2.9) | 16 (3.0) | 1 (1.7) | 5.9 |
| C09CA06 | Candesartan | 12 (2.1) | 11 (2.1) | 1 (1.7) | 8.3 |
| **J01** | **Antibiotics** | **76 (13.0)** | **62 (11.8)** | **14 (23.7)** | **18.4** |
| J01EE01 | Sulfamethoxazole-trimethoprim | 10 (1.7) | 8 (1.5) | 2 (3.4) | 20.0 |
| J01CR02 | Amoxicillin-clavulanic acid | 7 (1.2) | 6 (1.1) | 1 (1.7) | - |
| J01GB03 | Gentamicin | 6 (1.0) | 6 (1.1) | 0 (0) | - |

*The proportion of recorded AKIs is given for drugs involved in more than 10 cases of drug-induced AKI

Supplementary Table 6: Drug-induced CA-AKIs, according to the ATC classification level 2 drugs involved in more than 5% of cases, and the 3 most frequently involved drugs in each class

| **ATC classes** | **Drugs** | **All drugs suspected** | **Drugs suspected in the Cohort** | **Drugs suspected in the FPVD** | Proportion of AKIs recorded in the FPVD **(%)*** |
| --- | --- | --- | --- | --- | --- |
|  |  | **n=521** | **n=387** | **n=134** |  |
| **C03** | **Diuretics** | **125 (24.0)** | **101 (26.1)** | **24 (17.9)** | **19.2** |
| C03CA01 | Furosemide | 67 (12.9) | 60 (15.1) | 7 (5.2) | 10.4 |
| C03DA01 | Spironolactone | 28 (5.4) | 22 (5.7) | 6 (4.5) | 21.4 |
| C03AA03 | Hydrochlorothiazide | 18 (3.5) | 12 (3.1) | 6 (4.5) | 33.3 |
| **C09** | **Renin-angiotensin system drugs** | **91 (17.5)** | **82 (21.2)** | **9 (6.7)** | **9.9** |
| C09AA05 | Ramipril | 21 (4.0) | 19 (4.9) | 2 (1.5) | 9.5 |
| C09CA06 | Candesartan | 17 (3.3) | 15 (3.9) | 2 (1.5) | 11.7 |
| C09AA04 | Perindopril | 15 (2.9) | 12 (3.1) | 3 (2.2) | 20.0 |
| **L01** | **Antineoplastics** | **43 (8.3)** | **36 (9.3)** | **7 (5.2)** | **16.3** |
| L01XA01 | Cisplatin | 7 (1.3) | 7 (1.8) | 0 (0) | - |
| L01XA02 | Carboplatin | 6 (1.2) | 6 (1.6) | 0 (0) | - |
| L01BA01 | Capecitabin | 3 (0.6) | 3 (0.8) | 0 (0) | - |
| **A10** | **Antidiabetics** | **40 (7.7)** | **25 (6.5)** | **15 (11.2)** | **37.5** |
| A10BA02 | Metformin | 28 (5.4) | 21 (5.4) | 7 (5.2) | 25.0 |
| A10BH01 | Sitagliptin | 3 (0.6) | 2 (0.5) | 1 (0.7) | - |
| A10B09 | Gliclazide | 1 (0.2) | 0 (0) | 1 (0.7) | - |
| **B01** | **Antithrombotics** | 30 (5.8) | 25 (6.5) | 5 (3.7) | 16.7 |
| B01AF02 | Apixaban | 7 (1.3) | 6 (1.6) | 1 (0.7) | - |
| B01AC06 | Acetylsalicylic acid | 5 (1.0) | 3 (0.8) | 2 (1.5) | - |
| B01AC04 | Clopidogrel | 4 (0.8) | 3 (0.8) | 1 (0.7) | - |

* The proportion of recorded AKIs is given for drugs involved in more than 10 cases of drug-induced AKI
